# Extraintestinal Symptoms of Pain Are Common in Patients With Eosinophilic Gastrointestinal Diseases

**DOI:** 10.1016/j.gastha.2025.100732

**Published:** 2025-06-19

**Authors:** Jennifer Dziwis, Xiangfeng Dai, Chelsea Anderson, Ellyn Kodroff, Mary Jo Strobel, Amy Zicarelli, Sarah Gray, Amanda Cordell, Girish Hiremath, Evan S. Dellon, Elizabeth T. Jensen

**Affiliations:** 1 Department of Internal Medicine, Gastroenterology Section, Wake Forest University School of Medicine, Winston-Salem, North Carolina; 2 Center for Gastrointestinal Biology and Disease, Division of Gastroenterology and Hepatology, Department of Medicine, University of North Carolina School of Medicine, Chapel Hill, North Carolina; 3 Campaign Urging Research for Eosinophilic Disease (CURED), Lincolnshire, Illinois; 4 American Partnership for Eosinophilic Disorders (APFED), Atlanta, Georgia; 5 Eosinophilic Family Coalition (EFC), Dallas, Texas; 6 AusEE Inc, Frenchville, Australia; 7 EOS Network – Eosinophilic Diseases Charity, Colchster Essex, UK; 8 Division of Pediatric Gastroenterology, Hepatology, and Nutrition, Monroe Carell Jr. Children's Hospital at Vanderbilt, Vanderbilt University Medical Center, Nashville, Tennessee; 9 Center for Esophageal Diseases and Swallowing, University of North Carolina School of Medicine, Chapel Hill, North Carolina; 10 Department of Epidemiology and Prevention, Wake Forest University School of Medicine, Winston-Salem, North Carolina

**Keywords:** eosinophilic gastrointestinal diseases, extraintestinal pain, joint pain, headache

## Abstract

**Background and Aims:**

Extraintestinal symptoms are well-documented in systemic, inflammation-predominant conditions. Less is understood about extragastrointestinal symptoms among individuals with eosinophilic esophagitis (EoE) and non-EoE eosinophilic gastrointestinal diseases (EGIDs). We aimed to describe the differences in the frequency of patient-reported joint or leg pain and headache for EoE and non-EoE EGIDs individuals.

**Methods:**

Adult subjects and caregivers of children were recruited via the EGID Partners network and completed the Short-Form McGill Pain Questionnaire and Migraine Disability Assessment Test. T-tests were used to evaluate differences in extraintestinal pain symptomology by EGID type.

**Results:**

We analyzed 95 subjects with EoE only and 24 subjects with non-EoE only EGIDs. Both EoE and non-EoE EGID subjects described frequent leg pain (50% and and 78%, respectively). While no significant difference was observed in number of days of leg pain, pain severity was higher for non-EoE EGID subjects (*P* < .01). In addition, 78% of EoE only and 89% of non-EoE only EGID subjects reported joint pain, with no significant difference in the number of days of pain, but with higher pain severity for non-EoE EGID subjects (*P* < .01). Headache disability scores for non-EoE EGID subjects were more severe compared to subjects with EoE alone (17% of non-EoE EGIDs with severe disability vs 9% for EoE; *P* = .01).

**Conclusion:**

Patients with EGIDs may experience extraintestinal symptoms of pain. These symptoms may be more prominent in patients with non-EoE only EGIDs. Understanding of the underlying factors contributing to these symptoms is needed to guide mitigating approaches for these symptoms.

## Introduction

Eosinophilic gastrointestinal diseases (EGIDs) are a heterogenic group of diseases characterized by an inappropriately large quantity of eosinophils within specific areas of the gastrointestinal (GI) tract and associated GI symptoms, in the absence of secondary causes of eosinophilia.^1^ Eosinophilic esophagitis (EoE) is the most common and well described EGID with the less common subtypes being eosinophilic gastritis (EoG), eosinophilic enteritis (EoN), and eosinophilic colitis (EoC).^2^ Over the past 2 decades, the prevalence of EoE has risen dramatically, currently estimated to be 50 to 100 cases per 100,000 persons in United States.^3^, ^4^, ^5^ Similarly, the frequency of each of the nonesophageal EGIDs has been increasing.^6^^,^^7^ Patients suffering from EGIDs encounter significant health related costs driven by frequent health-care encounters, specialized diagnostic testing, and the need for lifelong medical therapies.^8^, ^9^, ^10^, ^11^

A well-recognized association between EGIDs and atopic disease exists, particularly for EoE and EoG, however the mechanism, thought to be at least in part driven by T helper cells and cytokine upregulation, remains widely debated.^12^, ^13^, ^14^ Likewise, mendelian-inherited connective tissue diseases (CTDs) including Marfan syndrome, Loeys-Dietz syndrome, and Eherlos Danlos may be prevalent in those with EoE. These diseases are driven by genetic variants involving the same signaling protein (Transforming Growth Factor Beta) that is upregulated in EoE pathogenesis, and the individuals affected by these conditions can have nearly 8-fold higher chance of having EoE compared to the general population.^15^ In addition, autoimmune conditions and CTDs have also been associated with EGIDs, including systemic sclerosis, lupus, rheumatoid arthritis, inflammatory myositis, and others, which have been identified as being more common in those with EGIDs.^16^, ^17^, ^18^, ^19^

Owing to EGIDs association with the aforementioned conditions and their complex pathophysiology, patients typically present with a wide array of GI symptoms. Depending on the area of eosinophilic infiltration in the GI tract, frequently reported gut symptoms include nausea, vomiting, abdominal pain, cramping, bloating, diarrhea, constipation, and weight loss. However, less is known about extraintestinal symptoms. Frequency and severity of gut, as well as certain extraintestinal symptoms, may be higher among those with EoG, EoN, or EoC compared to those with EoE only. For example, non-EoE EGID individuals may more commonly experience deep muscle or joint pain, fatigue, presyncope/syncope, flushing, and rashes,^8^ but these relationships are incompletely described. The prevalence of migraine has also shown to be higher in patients with GI disorders compared to the general population,^20^ though whether this is evident among those with EGIDs is not known. Therefore, we aimed to describe the differences in frequency and features of joint pain, leg pain, and headache among patients with EoE and non-EoE EGIDs.

## Methods

In this cross-sectional study, data were assembled from EGID Partners (egidpartners.org), an online patient-centered research network designed and implemented by patient advocacy groups and EGID researchers.^9^^,^^21^ Subjects were recruited via informational emails and through social media, directed messages to EGID patients through medical record patient portals, webinars, and by physicians. Participants self-reported diagnoses of EoE, EoG, EoN, or EoC, and we have previously shown that patient self-report of EGID diagnosis is reliable.^22^

Adult subjects and caregivers of children (<18 years) with an EGID completed surveys to document symptoms of leg and joint pain and the Short-Form McGill Pain Questionnaire ^23^ was used to assess affective (tension, fear, and autonomic response) and sensory (temporal, spatial, pressure, thermal) pain symptoms. This tool consists of 15 descriptors (11 sensory; 4 affective) which are rated on an intensity scale as 0 = none, 1 = mild, 2 = moderate, or 3 = severe. Three pain scores are calculated from the sum of the intensity values of the words chosen for sensory, affective, and total descriptors (maximum total score 45). In addition, subjects reported frequency of headaches, medication use for management of symptoms, and, among adults, severity of headache-associated disability using the Migraine Disability Assessment Test. This tool, comprised of 7 questions, generates a Migraine Disability Assessment Score (MIDAS) 0–21+ corresponding to degree with disability (0–5 = little or no disability, 6–10 = mild disability, 11–20 = moderate disability, 21+ = severe disability).^24^

For the present analysis, EGID subtypes were grouped into those reporting EoE only and those with EoG, EoN, or EoC, with or without concomitant EoE. Chi-square tests (for categorical variables) and two sample t-tests (for continuous variables) were used to evaluate differences in participants descriptive attributes, pain symptomology scores, including joint pain, leg pain, and migraine headache pain, according to the presence of EoE vs non-EoE EGID.

## Results

### Participant Demographic Characteristics by Eosinophilic Gastrointestinal Disease Type

A total of 119 EGID patients provided data regarding extra-GI symptoms. Most reported a diagnosis of EoE only (n = 95 vs n = 24 with EoG, EoN, and/or EoC with or without concomitant EoE). Most of the respondents self-identified as female (61%) and White race (93%) and most (86% for EoE only and 75% for non-EoE only EGIDs) were adults (≥18 years at diagnosis) (Table 1).

**Table 1:** Demographic and Clinical Attributes of Study Respondents

	EoE only (n = 95)	Non-EoE only EGID (n = 24)	*P*
Age at diagnosis - mean years ± SD	35.9 ± 18.2	37.8 ± 18.3	.65
Age <18 y (n, %)	14 (14)	6 (25)	.23
Sex, biologic at birth - n (%) (n = 84, 23)			.17
Female	49 (58)	17 (74)	
Male	35 (42)	6 (26)	
Gender - n (%) (n = 86, 23)			.49
Female	47 (55)	16 (70)	
Male	36 (42)	6 (26)	
Nonbinary	2 (2)	1 (4)	
Transgender	0 (0)	0 (0)	
Prefer not to respond	1 (1)	0 (0)	
Race - n (%) (n = 86, 23)			.65
American Indian or Alaskan Native	0 (0)	0 (0)	
Asian	2 (2)	1 (4)	
Black or African American	1 (1)	0 (0)	
Native Hawaiian or Other Pacific Islander	0 (0)	0 (0)	
White	79 (92)	22 (96)	
More than one race	4 (5)	0 (0)	
Prefer not to say	4 (5)	0 (0)	
Ethnicity - n (%) (n = 86, 23)			.36
Hispanic or Latino	3 (3)	0 (0)	
Not Hispanic or Latino	83 (97)	23 (100)	
Prefer not to say	0 (0)	0 (0)	
Symptom length prior to diagnosis (mean years ± SD)	8.2 ± 8.0	9.4 ± 8.2	.53
Symptoms (ever experienced) - n (%)			
Dysphagia (n = 84, 23 for all)	80 (95)	13 (57)	<.001
Food impaction	66 (79)	9 (39)	<.001
Heartburn	64 (76)	20 (87)	.27
Chest pain	36 (43)	12 (52)	.43
Abdominal pain	46 (55)	23 (100)	<.001
Nausea	48 (57)	22 (97)	.001
Vomiting	39 (46)	15 (65)	.11
Diarrhea	44 (52)	20 (87)	.003
Constipation	41 (49)	15 (65)	.16
Weight loss	26 (31)	17 (74)	<.001
Blood in stool	19 (23)	5 (22)	.93
Lower extremity edema	10 (12)	12 (52)	<.001
Atopic comorbidities (ever diagnosed) - n (%)			
Asthma (n = 86, 23 for all)	35 (41)	8 (35)	.61
Eczema	31 (36)	14 (61)	.03
Allergic rhinitis	66 (77)	13 (57)	.05
Food allergies	43 (50)	12 (52)	.85
Pollen food allergy syndrome	23 (27)	7 (30)	.73
Urticaria	28 (33)	8 (35)	.84

EGID, eosinophilic gastrointestinal disease.

There were no observed differences between age at diagnosis, sex distribution, race, or ethnicity between the EoE only and non-EoE only EGID groups. Patients in the EoE only group more commonly reported dysphagia (95% vs 57%; *P* < .01) and food impactions (79% vs 39%; *P* < .01) compared to the non-EoE only EGID group. Conversely, participants in the non-EoE only EGID group reported higher rates of abdominal pain (100% vs 55%; *P* < .01), weight loss (74% vs 31%; *P* < .01), and lower extremity edema (52% vs 12%; *P* < .01) (Table 1).

### Leg Pain

Participants with EoE and non-EoE only EGID reported suffering from leg pain >1 day per week on average, with no difference between groups (1.4 days vs 2.3 days; *P* = .09) (Table 2).

**Table 2:** Leg, Joint, and Headache Pain in EoE Only and Non-EoE Only EGIDs

	EoE only n = 95	Non-EoE only EGID n = 24	*P*^a^
Leg pain			
Days per week with pain in legs (mean ± SD) (n = 95, 24)	1.4 ± 2.2	2.3 ± 2.7	.09
Pain severity over prior 7 d (0–10 scale; mean ± SD) (n = 87, 24)	1.2 ± 1.8	3.6 ± 2.9	<.01
Short-Form McGill Pain Questionnaire (mean ± SD) (n = 78, 18)			
Total score	3.6 ± 5.0	11.8 ± 11.2	<.01
Sensory subscore	3.0 ± 4.0	9.5 ± 2.0	<.01
Affective subscore	0.6 ± 1.3	2.2 ± 2.7	<.01
Start of leg pain in relation to EGID symptoms - n (%)			<.01
Before	11 (14)	1 (6)	
After	20 (26)	5 (28)	
Same time	8 (10)	9 (44)	
N/A (no leg pain)	39 (50)	4 (22)	
Joint pain			
Days per week with pain in joints (mean ± SD) (n = 94, 24)	2.7 ± 3.0	3.8 ± 3.2	.12
Pain severity over prior 7 d (0–10 scale; mean ± SD) (n = 86, 19)	1.9 ± 2.0	4.6 ± 3.5	<.01
Short-Form McGill Pain Questionnaire (mean ± SD) (n = 81,18)			
Total score	4.1 ± 4.0	13.3 ± 10.7	<.01
Sensory subscore	3.5 ± 4.1	11.0 ± 8.5	<.01
Affective subscore	0.6 ± 1.1	2.3 ± 2.6	<.01
Start of joint pain in relation to EGID symptoms - n (%)			.39
Before	17 (22)	4 (22)	
After	31 (39)	8 (44)	
Same time	9 (11)	4 (22)	
N/A (no joint pain)	22 (28)	2 (11)	
Headache pain			
Days per week with headaches (mean ± SD) (n = 95, 24)	1.3 ± 1.8	2.1 ± 2.4	.06
Days of headaches in the last month (mean ± SD) (n = 94, 23)	4.5 ± 6.6	6.4 ± 9.3	.25
Take medications prescribed by a doctor for headaches - n (%) (n = 85, 22)	9 (11)	5 (23)	.13
Take over-the-counter medications for headaches - n (%) (n = 85, 22)	55 (65)	17 (77)	.26
Migraine disability assessment test (adults only) (n = 81, 18)			
Mean score (±SD)	7.1 ± 26.5	35.8 ± 81.8	.01
Grade of severity - n (%)			.01
I: Little or no disability (score 0–5)	66 (83)	9 (50)	
II: Mild disability (6–10)	3 (4)	1 (6)	
III: Moderate disability (11–20)	4 (5)	5 (28)	
IV: Severe disability (21+)	7 (9)	3 (17)	

EGID, eosinophilic gastrointestinal disease.

a Chi-square tests for differences in distribution of proportions and t-tests for testing differences in means.

Severity of leg pain was rated higher in the non-EoE only EGID group compared to the EoE only group (3.6 vs 1.2; *P* < .01). Individuals with non-EoE only EGIDs reported both significantly higher sensory as well as affective pain scores measured by the Short-Form McGill Pain Questionnaire (3 and 9.5, respectively, for sensory pain, 0.6 and 2.2 for affective pain; *P* < .01). Half of EoE only participants reported no leg pain while only 22% of non-EoE only EGID participants denied leg pain. If leg pain was reported, EoE only individuals were most likely to experience leg pain after their initial EoE symptoms (20%) where as non-EoE only EGID individuals were most likely to experience leg pain around the same time as their EGID symptoms developed (44%) (*P* < .01) (Table 2).

### Joint Pain

Participants with EoE only and non-EoE only EGID reported suffering from joint pain >2 days per week on average (2.7 days vs 3.8 days; *P* = .12) (Table 2). Severity of joint pain was higher in the non-EoE only EGID group compared to the EoE only group (4.6 vs 1.9; *P* < .01). Total pain scores from the McGill Pain Questionnaire were significantly higher for the non-EoE only EGID individuals compared to the EoE only individuals (13.3 vs 4.1, respectively; *P* < .01). This relationship persisted when scores are subdivided into sensory and affective pain scores (3.5 and 11, respectively, for sensory pain; 0.6 and 2.3 for affective pain for non-EoE only EGID compared to EoE only). There was no difference between the timeline of joint pain and GI symptoms when comparing non-EoE only EGID and EoE only patients (*P* = .39).

### Headache

Participants with EoE only and non-EoE only EGIDs reported suffering from headache >1 day per week on average (1.3 days vs 2.1 days, respectively; *P* = .06) (Table 2). The minority of individuals reported taking medication prescribed by a physician for headaches, however over half individuals noted over the counter medication use for alleviation of headache. As compared to those with EoE alone, adult individuals with non-EoE only EGID reported significantly higher scores on the Migraine Disability Assessment Test indicating greater disability (35.8 vs 7.1; *P* = .01). Specifically, 17% of non-EoE only EGID subjects indicated severe disability whereas only 9% of EoE only subjects noted severe disability (*P* = .01). There was no significant difference between the timeline of headache onset in relationship to EGID symptom onset when comparing non-EoE only EGID and EoE only patients (*P* = .62).

## Discussion

While “classic” symptoms attributable to the GI tract have been well described in EGIDs, the frequency of extra-GI symptoms is less well known. In this study, a high proportion of patients with either EoE alone or other EGIDs reported suffering from musculoskeletal pain and, on average, experienced >1 day per week with leg pain and >2 days/week with joint pain. Individuals with non-EoE only EGID rated their leg and joint pain as more severe and scored higher on the Short-Form McGill Pain Questionnaire than patients with EoE alone. We observed that leg pain was more likely to occur after development of initial EoE symptomatology whereas individuals with non-EoE only EGIDs developed leg pain earlier, or concurrently with other EGID symptoms. Headache frequency and perceived disability from migraine were also higher among individuals with non-EoE only EGID (severe disability) compared to EoE only.

Our findings of a higher prevalence of musculoskeletal pain among non-EoE only EGID individuals compared to EoE only individuals support results from a prior study that noted this trend.^8^ Our study expanded upon this observation by evaluating leg pain and joint pain independently while capturing valuable information on the severity and timing of onset of the pain (sensory and affective pain subtypes) utilizing a validated pain questionnaire.^23^

A key question, however, is why these extra-GI symptoms are present in what is thought to be a GI-predominant condition. Previous research has demonstrated an association between autoimmune and CTDs and EGID,^16^, ^17^, ^18^, ^19^ and these conditions are known to have multiple systemic symptoms. However, the presence of CTDs were not assessed in the present study and thus it is unknown whether the presence of these conditions could explain part or all of the associations observed for non-EoE EGID participants in our sample. Another possible explanation is the high frequency of patient-reported psychosocial symptoms among EGID patients.^8^^,^^25^, ^26^, ^27^, ^28^, ^29^ Common neuronal and systemic inflammation mechanisms are believed to underly physical and social pain,^30^, ^31^, ^32^ and it is therefore conceivable that a subset of non-EoE only EGID patients, who may be experiencing more pronounced GI-associated symptoms or more complex disease management approaches, may also experience increased feelings of isolation, and thus also increased frequency and severity of musculoskeletal pain and headache.^33^, ^34^, ^35^

A limitation to our study is its generalizability to all individuals with EGIDs as participants self-selected for enrollment in EGID Partners and participating in the surveys. In our study, patients self-reported their diagnosis of EGID and classified it as EoE, EoG, EoN, or EoC. While these diagnoses were not verified by the medical record, we previously reported on the high accuracy of this type of self-report in EGIDs.^22^ However, it is still possible that some individuals incorrectly reported an EGID diagnosis or misclassified their disease process. Another limitation of the study is its cross-sectional design. Study participants completed questionnaires electronically and the report of timing of pain symptom onset relative to EGID diagnosis may be subject to recall bias. Finally, the relatively low number of non-EoE EGID cases precludes examination of individual lower EGID diagnoses. Our study also has several strengths. Our study adds to the limited information regarding extraintestinal symptoms of EGIDs. Furthermore, we utilized the McGill Pain Questionnaire, a validated instrument, to assess details about the type, intensity and frequency of pain experienced, and the Migraine Disability Assessment Test, a validated instrument, to capture the impact of headache pain. This is also the first study, to our knowledge, to assess migraine as a potential extraintestinal manifestation of EGID.

## Conclusion

This study of the EGID Partners cohort found that extra-GI symptoms of leg pain, joint pain, and headaches are prevalent in patient with EGIDs, and that these symptoms are more prominent in patients with non-EoE only EGIDs. Understanding of the underlying factors contributing to these symptoms is needed to guide mitigating approaches for these symptoms. Direct measures of the incidence of inherited and autoimmune CTDs in individuals with EGIDs would be valuable in further understanding how these conditions may contribute to joint and musculoskeletal pain in EGID patients. In addition, headache may also be an important symptom to assess in this patient population, and when patients report non-GI symptoms in EGIDs, evaluation is needed to understand if there is a separate cause or if the symptom can be attributed to EGID, and whether psychologic factors may also be contributory.
